# ﻿Mammals in urban centers: a dataset from the perspective of the media in Brazil

**DOI:** 10.3897/zookeys.1223.129408

**Published:** 2025-01-15

**Authors:** Carolina Alves, Wellington Hannibal

**Affiliations:** 1 Laboratório de Ecologia e Biogeografia de Mamíferos, Universidade Estadual de Goiás, Avenida Brasil, Setor Helio Leao, Quirinópolis, GO, Brazil Universidade Estadual de Goiás Quirinópolis Brazil; 2 Programa de Pós-Graduação em Biodiversidade Animal, Universidade Federal de Goiás, Avenida Esperança, Chácaras de Recreio Samambaia, Goiânia, GO, Brazil Universidade Estadual de Goiás Quirinópolis Brazil; 3 Programa de Pós-Graduação em Ambiente e Sociedade, Universidade Estadual de Goiás, Avenida Brasil, Setor Helio Leao, Quirinópolis, GO, Brazil Universidade Estadual de Goiás Quirinópolis Brazil

**Keywords:** Carnivora, data paper, Ocelot, photographic records, São Paulo state, southeastern region

## Abstract

The continuous growth of the urban population, coupled with habitat loss, has resulted in unanticipated interactions between animals and humans in urban centers. In this study, we investigated the presence of mammals in urban centers through newspaper reports on websites. Specifically, we examined: i) the frequency of photographic records, ii) the temporal trends (2001 to 2021) and spatial trends (Brazilian Federative regions and states) of the records, and iii) the orders, families, and species most frequently reported in urban centers. On the Google platform (http://www.google.com.br), we used combinations of the keywords “mammals in urban centers,” “mammals found in the city”, and “mammals found in the municipality” to survey mammal records. We excluded repeated news items, sites that experienced technical problems during the search period, and those that did not cover the topic. We compiled a total of 733 websites. The records spanned from 2002 to 2021, with 73% occurring in the last four years. The Southeast, South, and Midwest regions stood out. The animals recorded belonged to 55 mammal species (16 vulnerable and 3 endangered), distributed in 22 families and 10 orders. The data indicate that the majority of mammal sightings in urban areas occur on streets, with some conflictual interactions. This is the first study that utilizes websites for diagnosing the mammal fauna present in urban centers in Brazil. The dataset generated here could aid in understanding the occurrence of mammal species in the urban environment.

## ﻿Introduction

Cities emerged thousands of years ago, and urban sprawl has led to a disruption in human-environment interaction (Seto et al. 2017; Perry et al. 2020). Continuous population growth and the demand for more resources alter and transform natural habitats, resulting in negative consequences for biodiversity (McDonald et al. 2013; Schenk and Souza 2014; Start et al. 2020); these consequences include the reduction of genetic diversity, threats from pathogens, the spread of exotic and invasive species, air, noise, and light pollution, as well as the alteration of natural hydrological regimes and fires (Theodorou 2022). Furthermore, population growth has been identified as one of the main causes of species and population extinction at a global level (Ceballos and Ehrlich 2002; Ceballos et al. 2010, 2015).

Ever since humans began living in settlements, wildlife has visited these places and found resources, such as shelters, food scraps, and garbage for food (Ceballos and Ehrlich 2002). As a result, there is an increase in the frequency of contact and complexity of the human-fauna relationship (Aronson et al. 2014). Species that were previously not observed in urban areas have been reported, even in cities with high population densities (Prezoto and Vale 2019). However, when wild species pose a threat to people and their livelihoods, this relationship can become conflictual (Zimmermann et al. 2010). In Brazil, conflicts between animals and humans have increased due to the migration of fauna from natural and rural areas to suburban and urban areas (Marchini and Crawshaw 2015).

Encounters and interactions between humans and animals have consequences for both. People are susceptible to zoonoses and economic damage, while animals face risks such as vehicle collision, entanglement, and attacks by domestic animals (Taylor-Brown et al. 2019). The frequency of recording wild animals in urban centers can be associated with local physical factors or the urban landscape, such as the presence of green areas, parks, waterways, and the often-practiced urban tree planting (Bateman and Fleming 2012; Van Bommel et al. 2020). Identifying these factors is important for formulating public policies and mitigating conflicts (Basak et al. 2020).

In this study, we investigated the presence of mammals in urban centers through newspaper reports and other communication networks on websites. Specifically, we examined: i) the frequency of photographic records, ii) the temporal trends (2001 to 2021), and spatial trends (Brazilian Federative regions and states) of records, and iii) the orders, families, and species most frequently reported in urban centers.

## ﻿Metadata

### ﻿Data set identity

**Title**: Mammals in urban centers: a dataset from the perspective of the media in Brazil.

**Data set identification code**: BRAZIL_SM_loc.csv, BRAZIL_SM_rec.csv, BRAZIL_SM_ref.csv, and BRAZIL_SM_int.csv.

### ﻿Data set description

**Principal investigators**:

Carolina Alves, Laboratório de Ecologia e Biogeografia de Mamíferos, Universidade Estadual de Goiás, Quirinópolis, GO, Brazil; carolinaalvesp97@gmail.com; ORCID: Carolina Alves (0000-0003-0501-3532).

Wellington Hannibal, Laboratório de Ecologia e Biogeografia de Mamíferos, Universidade Estadual de Goiás, Quirinópolis, GO, Brazil; wellingtonhannibal@gmail.com; ORCID: Wellington Hannibal (0000-0001-7141-1243).

### ﻿Overall project description

**Identity**: Compilation of mammals’ occurrence in urban centers, providing city, state and region of records, and richness, composition and threatened category data.

**Period of study**: The data presented were collected from 2002 to 2021, and the process of organizing and producing the current data set took place from 2021 to 2024.

**Objectives**: Our goal was to gather detailed information about mammal records in urban centers from Brazil, focusing on i) spatial trends of records (city, state and region); and ii) frequency in taxonomic records (orders, families and species) in urban centers of Brazil.

### ﻿Specific subproject description

**Site description**: Brazil is a country of continental proportions, with a territorial extension of 8,510,345.540 km^2^ (Instituto Brasileiro de Geografia e Estatística IBGE 2022), encompassing six climatic types: Equatorial, Semi-arid, Tropical, High-altitude Tropical, Atlantic Tropical, and Subtropical (Ministério do Meio Ambiente MMA 2022). Brazil is home to more than 100,000 animal species, encompassing mammals, birds, amphibians, fish, reptiles, insects, and invertebrates that inhabit forests, mangroves, savannahs, fields, rivers, and lakes across the following biomes: Amazon, Caatinga, Cerrado, Pantanal, Atlantic Forest, and Pampa (IBGE 2022, MMA 2022). The Brazilian mammal fauna consists of 778 species distributed across 11 orders, 51 families, and 247 genera (Abreu et al. 2022).

**Data collection**: The data were obtained from online media outlets, including news sites, videos, blogs, and government websites. We searched for potential studies in the following sources: (i) Google Search engine, (ii) social networks, (iii) newspapers, and (iv) government websites (city halls, state halls, and organizations such as the Fire Department and Military Police websites). We conducted a search for news stories using the following phrases: “mammals in urban centers”, “mammals found in the city”, “mammals seen in urban centers” and “mammals seen in the municipality” in Portuguese. Additionally, we employed a combination of keywords like the “common name of the species” (e.g., puma, capybara, monkey) along with the phrase “found in urban centers”, also in Portuguese.

**Research criteria**: We included in this database only news items that specifically reported the appearance of wild mammals in urban centers. From these sites, we extracted the following information: i) presence of a photo or video, ii) date, iii) city and state of the record, iv) geographic coordinates of the record and/or city, v) scientific name and main taxonomic categories (genus, family, and order), vi) name of the species reported on the site, and vii) title of the news item.

Taxonomic nomenclature was based on the updated checklist of Brazilian mammals by the Taxonomic Committee of the Brazilian Society of Mammalogy (Abreu et al. 2022). We identified the species using field guides and books on mammals in Brazil, as well as the species' distribution areas according to the IUCN Red List. (Bonvicino et al. 2008; Reis et al. 2011; Nascimento and Feijó 2017; Faria et al. 2019; Azevedo et al. 2021; Menezes et al. 2021; Rumiz et al. 2022; IUCN 2022). We added a column with the current scientific name based on the aforementioned literature. However, due to the lack of a photo or video, the poor quality of the image or footage, and the existence of a species complex for the same genus at the cited site, some species were identified only at the genus level, followed by “sp.” or “spp.” In these cases, we filled in the cell in the ‘Actual_species_name’ column with the genus, followed by “sp.”.

### ﻿Data set status and accessibility

**Data verification**: All localities were checked for accuracy and precision. The taxonomic status of the species was verified by the authors. In the bibliographic records, the taxonomic update was made based on the most recent literature. Carolina Alves conducted the searches and analysis of websites for inclusion in this dataset, carefully evaluating which ones met the inclusion criteria. Wellington Hannibal analyzed the dataset and created the figures. The data were mostly derived from news websites and newspapers, and we sought to correct any errors in taxonomic information about the species.

### ﻿Accessibility

**Storage location and medium**: Available as Supporting Information to this Ecology Data Paper in .csv format (https://figshare.com/articles/dataset/_b_MAMMALS_IN_URBAN_CENTERS_a_dataset_for_Brazil_b_/26616214).

**Contact person**: Wellington Hannibal, Laboratório de Ecologia e Biogeografia de Mamíferos, Universidade Estadual de Goiás, Quirinópolis, Goiás, 75860-000, Brazil. E-mail: wellingtonhannibal@gmail.com

**Copyright restrictions**: None

**Proprietary restrictions**: Please cite this data paper when using it in publications. We also request that researchers and teachers inform us of how they are using the data.

**Costs**: None.

### ﻿Data set file

BRAZIL_SM_loc.csv

BRAZIL_SM_rec.csv

BRAZIL_SM_ref.csv

BRAZIL_SM_int.csv

**Format and storage mode**: comma-separated values (.csv).

**Header information**: See Table 1 in section B for column descriptions.

**Table 1.** Description of columns of .csv files.

**BRAZIL_SM_loc.csv**	
**id**	Code given to each locality
**Municipality**	Municipality of the locality
**State**	State of the locality
**Lat**	Decimal coordinates of the locality
**Long**	Decimal coordinates of the locality
**Datum**	Geodetic coordinate system
**Coordinates Location**	Reference from where the coordinates were obtained
**Biomes**	Biomes from where the coordinates were obtained
**BRAZIL_SM_rec.csv**	
**id**	Code given to each locality
**Month**	Month when the record was published
**Year_Publication**	Year when the record was published
**Order**	Order taxonomic classification
**Family**	Family taxonomic classification
**Genus**	Genus taxonomic classification
**Species_name_on_site**	Species name published on website
**Actual_species_name**	Species name according taxonomic classification
**Species_origin**	Origin of species
**Record_Type**	Type of record, photography, video
**BRAZIL_SM_ref.csv**	
**id**	Code given to each locality
**Site_Name**	Name of the site where record was published
**Type_Site**	Category of the site where the record was published
**Link**	Link to website
**BRAZIL_SM_int.csv**	
**id**	Code given to each locality
**Location**	Exact location where the animal was found
**Rescueorganization**	Agency responsible for the rescue
**Destination**	Release or sent for rehabilitation
**Interactions**	Whether there was human-wildlife interaction
**Injuries**	Whether there was an injury or not
**Zone**	Encounter in rural, urban, or peri-urban area
**deceased**	The animal died

### ﻿Tables and figures

Table 1. Description of columns of .csv files;

Table 2. Systematic list of mammals’ species in urban areas of Brazil.

**Table 2.** Systematic list of mammal species in urban areas of Brazil. Brazilian states legend: Acre (AC), Alagoas (AL), Amapá (AP), Amazonas (AM), Bahia (BA), Ceará (CE), Distrito Federal (DF), Espírito Santo (ES), Goiás (GO), Maranhão (MA), Mato Grosso (MT), Mato Grosso do Sul (MS), Minas Gerais (MG), Pará (PA), Paraíba (PB), Paraná (PR), Pernambuco (PE), Piauí (PI), Rio de Janeiro (RJ), Rio Grande do Norte (RN), Rio Grande do Sul (RS), Rondônia (RO), Roraima (RR), Santa Catarina (SC), São Paulo (SP), Sergipe (SE), Tocantins (TO).

Taxon	Common Name	Federative Unit
**Didelphimorphia Gill, 1872**		
**Didelphidae Gray, 1821**		
*Caluromysphilander* (Linnaeus, 1758)	Bare-tailed Woolly Opossum	ES
*Didelphisalbiventris* Lund, 1840	White-eared Opossum	DF, MG, MS, PR, RS, SC, SP
*Didelphisaurita* (Wied-Neuwied, 1826)	Big-eared Opossum	ES, MG, RJ, RS, SC, SP
*Philandercanus* (Osgood, 1913)	Gray Four-eyed Opossum	GO
**Cingulata Illiger, 1811**		
**Chlamyphoridae Bonaparte, 1850**		
*Euphractussexcinctus* (Linnaeus, 1758)	Six-banded Armadillo	ES, MS, TO
*Cabassoustatouay* (Desmarest, 1804)	Southern Naked-tailed Armadillo	RJ
*Priodontesmaximus* (Kerr, 1792)	Giant Armadillo	TO
*Tolypeutesmatacus* (Desmarest, 1804)	Southern Three-banded Armadillo	MS
*Tolypeutestricinctus* (Linnaeus, 1758)	Brazilian Three-banded Armadillo	CE
**Dasypodidae Gray, 1821**		
*Dasypusnovemcinctus* Linnaeus, 1758	Nine-banded Armadillo	AC, MG, MS, PR, RJ, RS
**Pilosa Flower, 1883**		
**Bradypodidae Gray, 1821**		
Bradypus (Scaeopus) crinitus Gray, 1850	Maned Three-toed Sloth	RJ
Bradypus (Bradypus) variegatus Schinz, 1825	Brown-throated Three-toed Sloth	AM, BA, CE, MG, PE, RJ, SC, SP
**Myrmecophagidae Gray, 1825**		
*Myrmecophagatridactyla* Linnaeus, 1758	Giant Anteater	GO, MG, MS, MT, RR, SP, TO
*Tamanduatetradactyla* (Linnaeus, 1758)	Southern Tamandua	AM, AP, BA, CE, ES, MG, MS, MT, PR, RJ, RN, RS, SC, SP, TO
**Primates Linnaeus, 1758**		
**Atelidae Gray, 1825**		
*Alouattacaraya* (Humboldt, 1812)	Black-and-gold Howler Monkey	GO, MS, RS
*Alouattaguariba* (Humboldt, 1812)	Brown Howler Monkey	MG, PR, RJ, RS, SC, SP
**Cebidae Bonaparte, 1831**		
*Callithrixaurita* (É. Geoffroy St.-Hilaire, 1812)	Buffy-tufted-ear Marmoset	RJ
*Callithrixpenicillata* (É. Geoffroy St.-Hilaire, 1812)	Black-pencilled Marmoset	MG, PR
*Saimiricollinsi* Osgood, 1916	American Squirrel Monkey	MA
**Rodentia Bowdich, 1821**		
**Caviidae Fischer, 1817**		
*Hydrochoerushydrochaeris* (Linnaeus, 1766)	Capybara	DF, ES, GO, MS, MT, PE, RJ, RN, RS, SC, SE, SP, TO
**Cuniculidae G. S. Miller & Gidley, 1918**		
*Cuniculuspaca* (Linnaeus, 1766)	Lowland Paca	GO, MG, PR
**Dasyproctidae Bonaparte, 1838**		
*Dasyproctaazarae* Lichtenstein, 1823	Azara’s Agouti	MS
*Myoproctapratti* Pocock, 1913	Green Acouchi	AM
**Dinomyidae Alston, 1876**		
*Dinomysbranickii* Peters, 1873	The Pacarana	AC
**Echimyidae Gray, 1825**		
*Myocastorcoypus* (Molina, 1782)	Coypu, Nutria, River rat. The Nutria	PR, RS
**Erethizontidae Bonaparte, 1845**		
*Chaetomyssubspinosus* (Olfers, 1818)	Bristle-spined Rat	BA
*Coendouprehensilis* (Linnaeus, 1758)	Brazilian Porcupine	CE, DF, MG, MS, PR, RJ, RO, RS, SC, SP, TO
*Coendouspinosus* (Cuvier, 1823)	Paraguaian Hairy Dwarf Porcupine	ES, MG, RJ, RS, SP
**Sciuridae Fischer, 1817**		
*Guerlinguetusaestuans* (Linnaeus, 1766)	Guianan Squirrel	RS
*Guerlinguetusbrasiliensis* (Gmelin, 1788)	Ingram’s squirrel	BA, PR, RJ, SC
**Carnivora Bowdich, 1821**		
**Canidae Fischer, 1817**		
*Cerdocyonthous* (Linnaeus, 1766)	Crab-eating Fox	BA, CE, DF, ES, MA, MG, MS, PR, RJ, RS, SC, SE, SP
*Chrysocyonbrachyurus* (Illiger, 1815)	Maned Wolf	GO, MG, MS, MT, PI, PR, RJ, SP, TO
*Lycalopexvetulus* (Lund, 1842)	Hoary Fox	GO, MG, SP, TO
*Lycalopexgymnocercus* (Fischer, 1814)	The Pampas Fox	RS
*Speothosvenaticus* (Lund, 1842)	Bush Dog	MS, MT
**Felidae Fischer, 1817**		
*Herpailurusyagouaroundi* (É. Geoffroy Saint-Hilaire, 1803)	Jaguarundi	BA, CE, DF, MG, MS, MT, PA, PE, RS
*Leopardusguttulus* (Hensel, 1872)	Southern Tiger Cat	ES, MG, PR, RS, SC, SP
*Leoparduspardalis* (Linnaeus, 1758)	Ocelot	AC, AL, BA, CE, ES, GO, MG, MS, MT, PA, PB, PE, PI, PR, RJ, RS, SC, SE, SP, TO
*Leopardustigrinus* (Schreber, 1775)	Little Spotted Cat	CE, PB
*Leoparduswiedii* (Schinz, 1821)	Margay	AL, AM, AP, MA, PR, RS, SC, SP
*Pumaconcolor* (Linnaeus, 1771)	Puma	BA, ES, GO, MG, MS, MT, PA, PR, RJ, SC, SP, TO
*Pantheraonca* (Linnaeus, 1758)	Jaguar	AL, AM, GO, MG, MS, MT, PR, RR, SP, TO
**Mustelidae Fischer, 1817**		
*Eirabarbara* (Linnaeus, 1758)	Tayra	RS
*Galictiscuja* (Molina, 1782)	Lesser Grison	MG, PR, RS, SC, SE, SP
*Pteronurabrasiliensis* (Zimmermann, 1780)	Giant Otter	AM, TO
*Lontralongicaudis* (Olfers, 1818)	River Otter	AP, BA, MS
**Procyonidae Gray, 1825**		
*Nasuanasua* (Linnaeus, 1766)	South American Coati	BA, ES, MG, MS, MT, PB, PE, PR, RJ, RS, SP
*Potosflavus* (Schreber, 1774)	Kinkajou	RJ, RO
*Procyoncancrivorus* (Cuvier, 1798)	Crab-eating Raccoon	MG, MT, RS, SC, SP
**Perissodactyla Owen, 1848**		
**Tapiriidae Gray, 1821**		
*Tapirusterrestris* (Linnaeus, 1758)	Lowland Tapir	MG, MS, MT, SP
**Cetartiodactyla Montgelard, Catzeflis & Douzery, 1997**		
**Cervidae Goldfuss, 1820**		
*Mazamarufa* (Erxleben, 1777)	Red Brocket	DF, ES, MG, MS, MT, SP, TO
*Subulogouazoubira* (Fischer, 1814)	Gray Brocket	BA, CE, ES, GO, MG, MS, MT, PR, RS, SC, SP, TO
**Tayassuidae Palmer, 1897**		
*Dicotylestajacu* (Linnaeus, 1758)	Collared Peccary	MT, TO
*Tayassupecari* (Link, 1795)	White-lipped Peccary	MS

**Figure 1.**
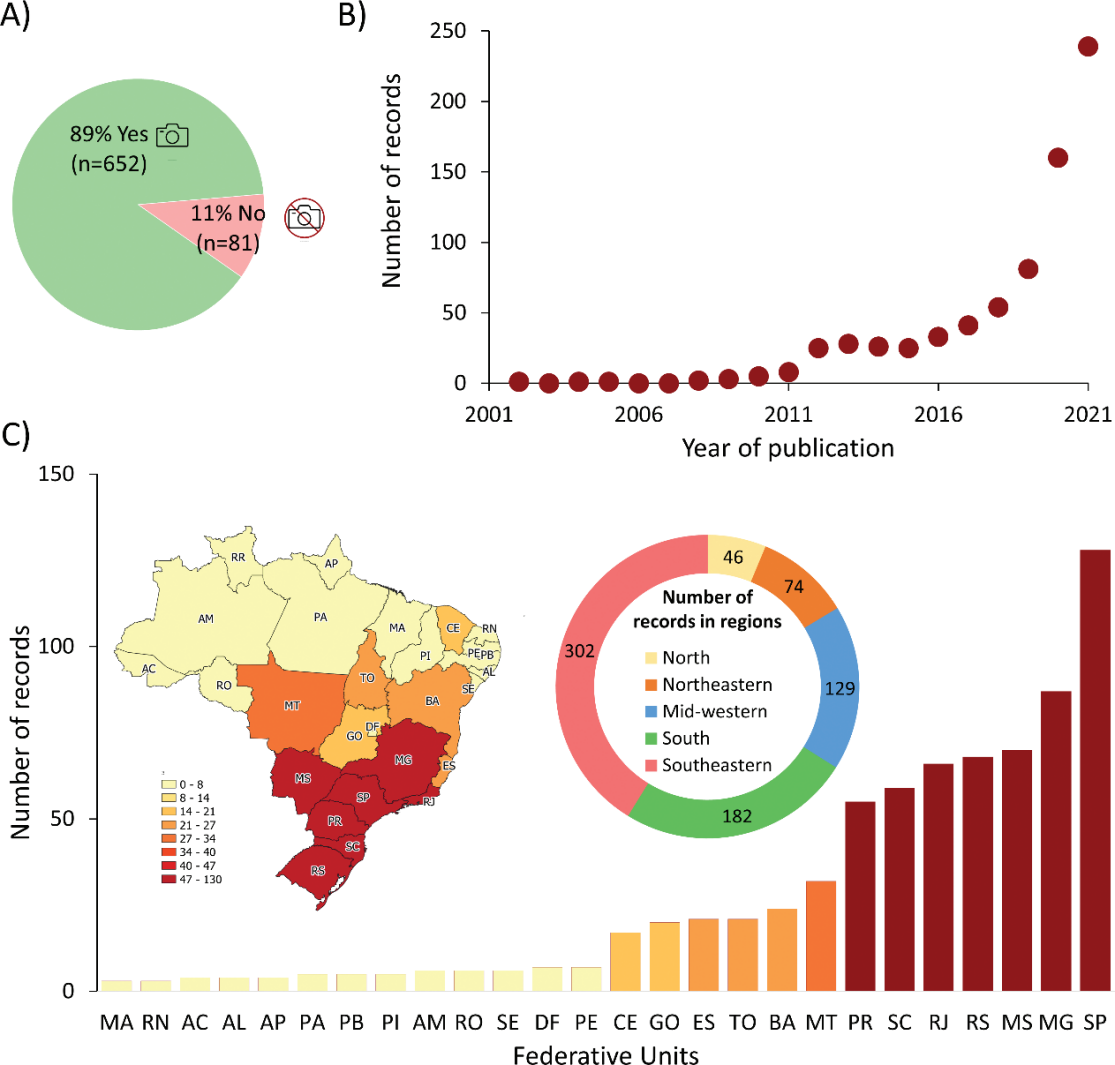
Number of photographic (**A**), temporal (**B**) and spatial (**C**) records of mammalian species in urban areas of Brazil.

**Figure 2.**
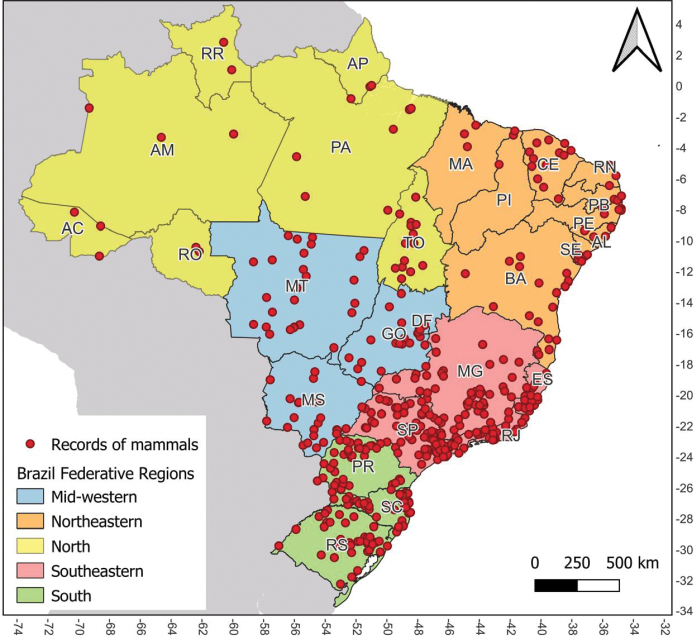
Geographic distribution of mammal occurrence records in Brazilian urban areas, categorized by federative regions.

**Figure 3.**
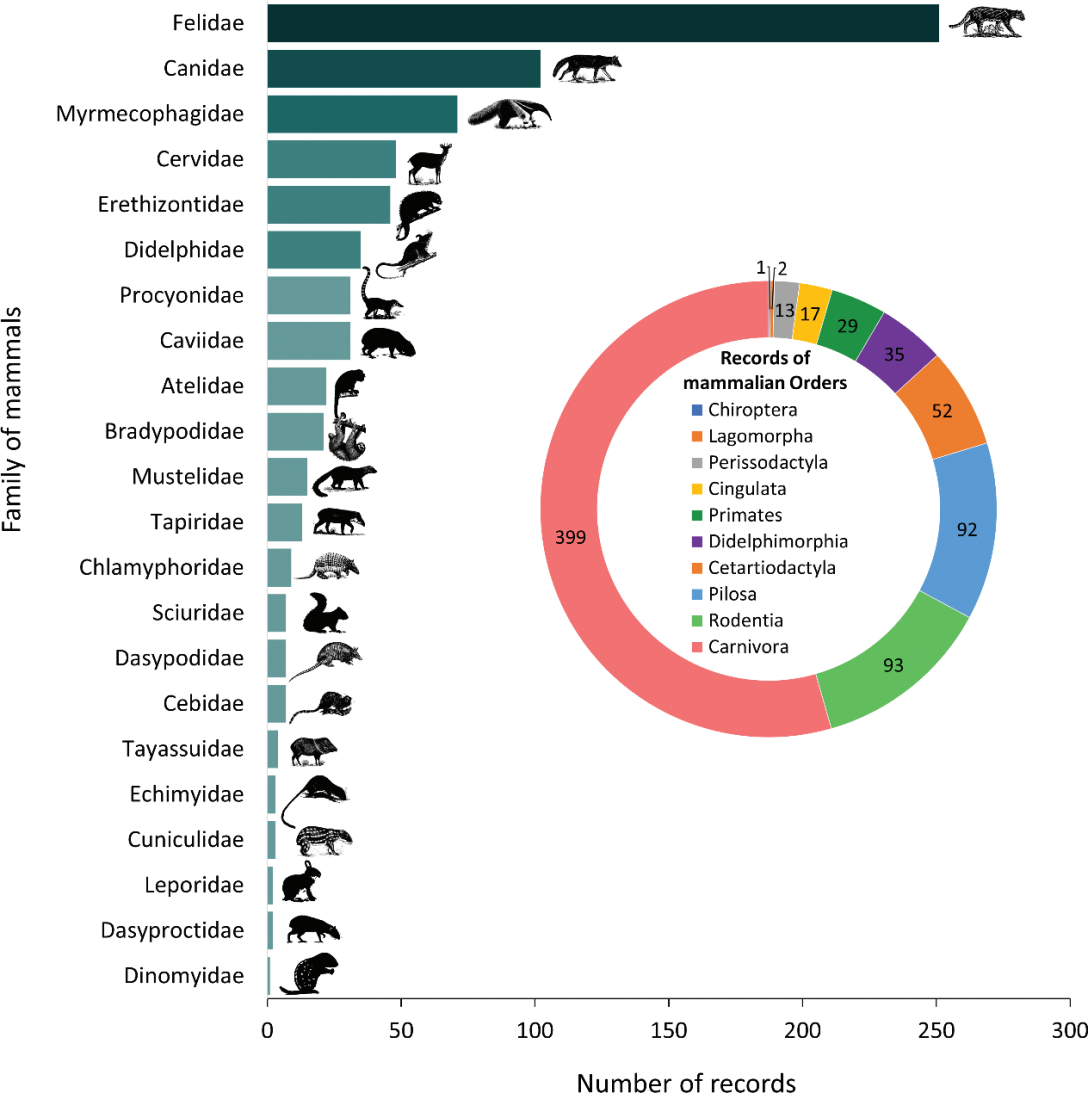
Number of records by orders (pie plot) and families (bar plot) of mammals in urban areas of Brazil.

**Figure 4.**
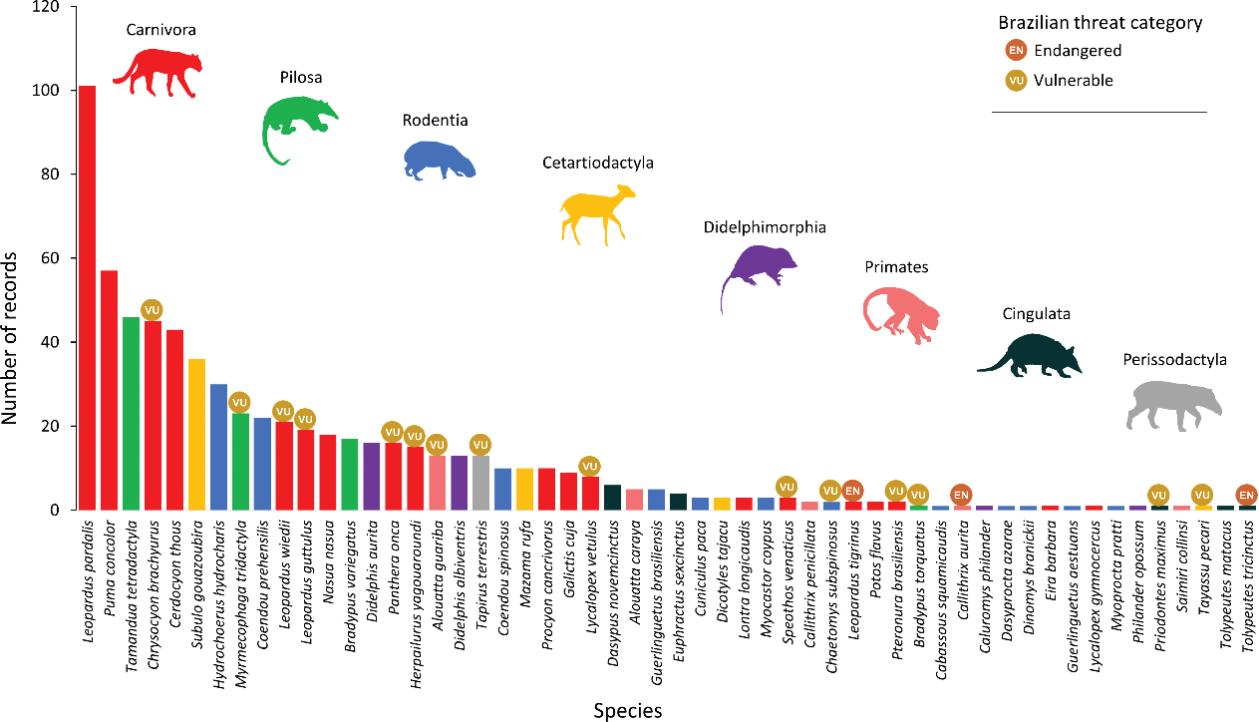
Number of records by species of mammals in urban areas of Brazil.

**Figure 5.**
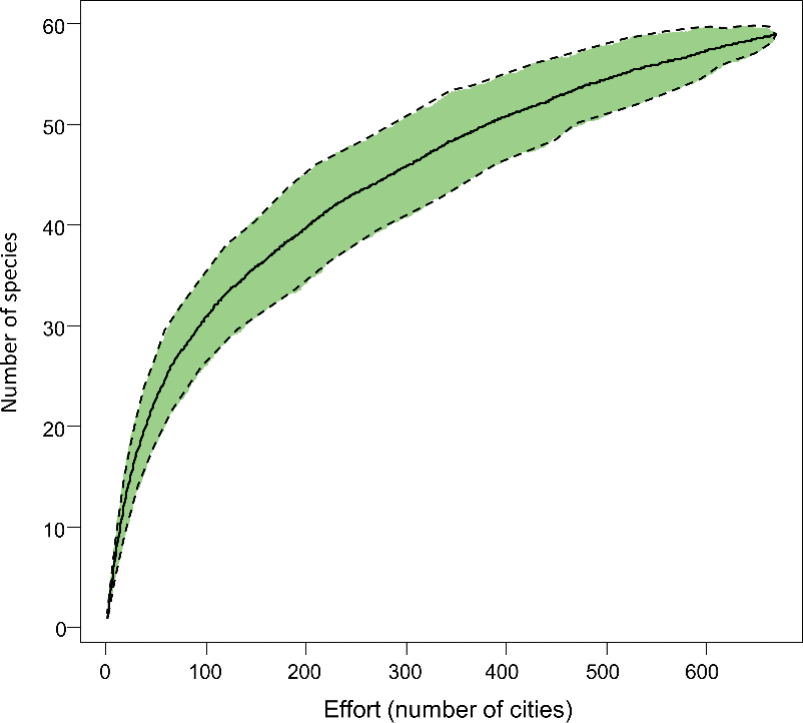
Collector’s curve showing species accumulation with increasing sampling effort across urban areas.

Figure 1. Number of photographic, temporal and spatial records of mammalian species in urban areas of Brazil;

Figure 2. Geographic distribution of mammal occurrence records in Brazilian urban areas, categorized by federative regions;

Figure 3. Number of records by families of mammals in urban areas of Brazil;

Figure 4. Number of records by species of mammals in urban areas of Brazil;

Figure 5. Collector’s curve showing species accumulation with increasing sampling effort across urban areas.

## ﻿Results description

This dataset comprises 733 records of 450 mammal locations found in urban centers across Brazil, as reported on various websites. Of the total number of records, 89% (*N* = 652) included an image or video, spanning the period between 2002 and 2021, with a noticeable increase in the number of records in the last five years (Fig. 1). The Southeast (41%, *N* = 302), South (25%, *N* = 182), and Midwest (18%, *N* = 129) regions had the highest number of records, particularly in the cities of São Paulo, Minas Gerais, Mato Grosso do Sul, Rio Grande do Sul, Rio de Janeiro, Santa Catarina and Paraná (Figs 1, 2).

Our data demonstrate a geographic bias in media reports on human-mammal encounters in urban areas (Figs 1, 2). The regions showing the highest number of records are economically more developed compared to other areas of the country (Saraiva, Souza, 2012). Consequently, these regions have greater media coverage. One recommendation to reduce this bias is to foster stronger communication between scientists and the media, along with more studies to investigate public perception of wildlife and interpretation of media events. Additionally, increased investment in communication, education, and public awareness programs could help rebalance both media and public perception (Bornatowski et al. 2019).

Of the total 733 records, we found 55 species, 22 families and 10 orders of mammals in urban areas of Brazil (Table 2). Carnivora (*N* = 399 records, 19 species) was the more representative order, followed by Rodentia (93, 11 spp.), Pilosa (92, 4 spp.), Cetartiodactyla (52, 4 spp.), Didelphimorphia (35, 4 spp.), Primates (29, 5 spp.), Cingulata (17, 6 spp.), Perissodactyla (13, 1 sp.), Lagomorpha (2, 1 sp.) and Chiroptera (1, 1 sp.). Felidae and Canidae comprised 48% of records (*N* = 353); Felidae occur in 96% of localities (Fig. 3).

The Ocelot, Puma, Southern Tamandua, Maned Wolf, Crab-eating Fox, Gray Brocket, and Capybara reach more than 30 records and represented 49% of mammalian fauna in urban areas from Brazil (Fig. 4). Of the total species recorded in urban areas, 32.7% are threatened according Brazilian Red List (MMA 2022), highlighted by the orders: Carnivora (Maned Wolf *Chrysocyonbrachyurus*, Hoary Fox *Lycalopexvetulus*, Bush Dog *Speothosvenaticus*, Margay *Leoparduswiedii*, Southern Tiger Cat *L.guttulus*, Jaguar *Pantheraonca*, Jaguarundi *Herpailurusyagouaroundi* and Giant Otter *Pteronurabrasiliensis*), Cingulata (Giant Armadillo *Priodontesmaximus* and Brazilian Three-banded Armadillo *Tolypeutestricinctus*), Pilosa (Giant Anteater *Myrmecophagatridactyla* and Maned Three-toed Sloth *Bradypustorquatus*), Primates (Brown Howler Monkey *Alouattaguariba* and Buffy-tufted-ear Marmoset *Callithrixaurita*), Perissodactyla (Lowland Tapir *Tapirusterrestris*) and Cetartiodactyla (White-lipped Peccary *Tayassupecari*) (Fig. 4)

The species accumulation curve (Fig. 5) provides validation for using this dataset to make inferences about mammal diversity in urban areas within the sampled context. The curve shows a gradual plateau, indicating that a sufficient sampling effort (in terms of the number of cities) has been reached to capture the diversity most frequently reported in the media. However, we acknowledge that the data carry an inherent media bias, favoring reports of mammals that capture public attention—typically emblematic, charismatic, and vulnerable species more likely to be impacted by human activities. This is because, for an event to become newsworthy, it must hold relevance from the media’s perspective, drawing public attention (Freitas and Barszcz 2015; Shaw et al. 2022).

Thus, the media focus on these specific species is a reflection of journalistic trends rather than a methodological flaw in the study. Although this bias may prevent uniform records across all species, the accumulation curve suggests that the data collected still provide a legitimate basis for understanding broader trends. It serves as a valuable repository of information on the increasing frequency of human-wildlife interactions in urban areas, opening pathways for further discussions on how media coverage influences public perception of urban wildlife. While this dataset may not fully reflect the actual diversity or abundance of species in urban areas, it highlights patterns in human-wildlife relationships shaped by media representation, offering an opportunity for future analyses of these dynamics.

Based on reports gathered through the media, the locations with the highest number of mammal sightings in urban centers are streets (280 records), followed by residential properties (191), highways (116), parks (41), businesses (33), gated communities (19), vacant lots (9), schools (5), hospitals (5), airports (2), churches (2), rivers (2), hotels (1), banks (1), nursing homes (1), universities (1), and gardens (1). Other reports did not specify where the animals were sighted or found. After being located, 292 records indicate that the mammals were sent for rehabilitation, with 188 of these animals found injured and 99 fatalities recorded.

Regarding interactions, not all reports included information on conflicts or relationships beyond encounters between humans and wildlife. The recorded interactions include road accidents (108), conflicts with dogs (16), predation of domestic animals (10), retaliation (4), electric shocks (3), intentional feeding (3), poisoning (2), nuisance wildlife (2), mutilation (1), and crop damage (1). Interactions between humans and wild animals, particularly mammals, are diverse and complex, often resulting in conflicts. Both habitat loss due to urbanization and agricultural expansion, along with the presence of urban parks, contribute to these conflicts (Griffin et al. 2022; Adhikari et al. 2024). The majority of records (679) are from urban areas, as the database focuses on mammals in urban centers.

## ﻿Final considerations

Compiling information on all mammal species found in urban centers into a single document is particularly challenging for several reasons: i) sites with incomplete information, ii) incorrectly identified animals, iii) sites with technical problems, and iv) poor-quality photos and videos.

Even so, our dataset reflects the number of mammal records in urban centers in Brazil. This is the first study to utilize websites to diagnose the mammal fauna present in urban centers in Brazil. The dataset generated here could help us understand the occurrence of mammal species in urban environments and serve as a foundation for future studies related to urban landscape ecology and its implications for the distribution and conservation of mammals in these environments.

## ﻿Additional information

### Author ORCIDs

Carolina Alves https://orcid.org/0000-0003-0501-3532

Wellington Hannibal https://orcid.org/0000-0001-7141-1243

### Data availability

Data published through figshare: https://figshare.com/articles/dataset/_b_MAMMALS_IN_URBAN_CENTERS_a_dataset_for_Brazil_b_/26616214.
